# Navigating in the fog. Facing delays, rejection and ignorance when seeking help for primary hyperhidrosis

**DOI:** 10.1080/17482631.2021.1930642

**Published:** 2021-05-31

**Authors:** Alexander Shayesteh, Fredrik Gerdsdorff, Margareta Persson, Christine Brulin, Elisabet Nylander

**Affiliations:** a Department of Public Health and Clinical Medicine, Dermatology and Venereology, Umeå University, Umeå, Sweden; b Department of Nursing, Umeå University, Umeå, Sweden

**Keywords:** Primary hyperhidrosis, care givers, healthcare, experience of healthcare, qualitative research

## Abstract

Primary hyperhidrosis (PH) is a disease characterized by focal and excessive sweating.

**Purpose**: The aim of this study was to describe the experiences of men and women with PH when seeking help for their condition.

**Method**: A qualitative interview study with 30 men and women diagnosed with PH was conducted. Data was inductively analysed using manifest and latent content analysis.

**Results**: The analysis resulted in a theme: *Navigating in the fog*, based on the categories *doubtful encounters with health care professionals, helpful encounters with health care professionals, delays due to inadequate knowledge*, and *supported urge for help*.

**Conclusions**: Deficient knowledge and understanding about PH create a sense of resignation in individuals, resulting in delay of seeking treatment. Support from others, life-changing events, and finding information about PH were important motivating factors in seeking help and demanding access to treatment. A holistic approach towards patients with PH is important to reduce stigma and acknowledge the problems that are encountered in their daily lives. Educating health care professionals and students so that patients can be identified and assessed without delay and making information available about PH in schools and pharmacies could improve the general knowledge and facilitate obtaining treatment for individuals with PH.

## Introduction

Hyperhidrosis is defined as excessive sweating more than is physiologically required for thermoregulation. Primary hyperhidrosis (PH) is defined as idiopathic and focal excessive sweating (Stolman, 2003). In a cross-sectional survey, the prevalence of PH was estimated at 5.5% in Sweden, affecting both sexes in equal proportions (Shayesteh et al., 2016). Primary hyperhidrosis starts in adolescence, and the sweating is most common in the armpits (Doolittle et al., 2016).The diagnosis of PH is based on the patient’s medical history, while the severity of sweating can be assessed using the Hyperhidrosis Disease Severity Scale (HDSS) (Hornberger et al., 2004). The HDSS contains one question with a choice of four answers from which the patients should choose one; a score of three or four points indicates severe hyperhidrosis (Kowalski et al., 2004).

First-line treatment in PH consists of over-the-counter deodorants containing aluminium salts compounded with alcohol (Solish et al., 2007). Topical remedies are easy to obtain from stores and pharmacies; however, in severe cases of PH, the patient must either be referred or seek access to more costly treatments such as botulinum toxin A injections. Botulinum toxin is injected into the skin, inhibiting nerve impulses to the sweat glands and diminishing sweat production (Naumann & Lowe, 2001). Although very effective, treatment must be repeated once or twice per year to maintain its effect. Systemic anticholinergics may also be used to reduce sweat production, with risk of side effects such as dry mouth and gastrointestinal symptoms (Hornberger et al., 2004). Surgery is mostly reserved as a last resort due to potential and severe complications (Stolman, 2003).

PH has a negative impact on the social life of those affected (Lenefsky & Rice, 2018). The consequences of PH, affecting quality of life compared to other dermatological diseases, have been reported to be more extensive than acne, eczema, and psoriasis (Kjeldstrup Kristensen et al., 2020) .The daily interferences of excessive sweating by staining cloths, leaving marks on papers, and being exposed to social interaction when the sweating is revealed, have been associated with decrease in mental health by creating a sense of shame and stigmatizing the individual (Kamudoni et al., 2017; Shayesteh et al., 2019). While more qualitative research is warranted in adults with PH, a study in 10 adolescents aged 12–18 years, described that stigma associated with PH creates an obsession in striving for an ideal, challenged by the individuals view of normality (Trettin et al., 2020). It was also reported that adolescents with PH, neglected by general practitioners, were told that their excessive sweating represented a physiological part of adolescence.

Individuals with PH seldom seek medical help for their sweat problems. Being unaware of existing treatments and not recognizing the symptoms have been described as possible reasons (Doolittle et al., 2016). Descriptions regarding the process of seeking help in PH are scarce, and there is a need of more knowledge about this relatively unknown disease. Thus, we conducted a qualitative study with the aim of exploring the experiences of men and women with PH when seeking help for their condition.

## Method

### Aim

To describe the experiences of men and women with PH when seeking help.

### Study design

An inductive interview design comprising individual interviews was applied to the study, and qualitative content analysis was used for analysis of the experiences from 15 men and 15 women seeking help for PH.

### Settings and procedure

Patients with PH referred by primary health care were recruited consecutively from the clinic for hyperhidrosis within the Department of Dermatology and Venereology, Umeå University Hospital, during 2016 and 2017. Purposive sampling was used, and patients who had PH for many years were invited to participate. The inclusion criterion was PH with HDSS >2 points. Exclusion criteria were (1) individuals absent of a main (most affected) sweating site (2) multifocal hyperhidrosis (i.e., >2 sweating sites), and (3) received treatment with botulinum toxin prior to the interview (one-year minimum). In the recruiting process, 30 men and 24 women were asked for their participation; however, 22 individuals declined due to their life situation, work, and travelling distances, while 2 individuals were excluded because of multifocal hyperhidrosis. The diagnosis of primary hyperhidrosis in all participants had been confirmed by the general practitioner and verified by a dermatologist. A total of 15 men and 15 women participated in this study (Table I).

**Table I.** Characteristics of the participants presented as median (md) or number (n)

Participants	Men (n = 15)	Women (n = 15)
Age (md)	32	26
Age at onset (md)	14	13
Marital status (n): *Married* *Partnership* *Single*	2 8 5	3 5 7
Occupation (n): *Light physical work* *Heavy physical work*	10 5	13 2
Hyperhidrosis (n): *Axillary* *Palmar* *Plantar* *Axillary and palmar*	7 4 1 3	7 3 1 4

### Data collection

The interviews focused on the participants’ experiences of information seeking, motivation to seek help, and their path in health care for obtaining treatment. Based on the topic of this study, an interview guide was developed according to our clinical experience and known literature, and used as a support during the interviews. The participants were asked to describe their experiences, which were illustrated with vignettes. These experiences consisted of actual situations or problems that were known to often cause concerns in contact with others or by themselves (Sampson & Johannessen, 2020). Moreover, to follow up interesting and important topics, the interviewer also used probing questions such as “What do you mean by … ?”, “Could you please elaborate … ?” and “Such as?”. The interviews lasted for 40–50 minutes. All interviews were performed by the first author (AS) at Umeå University Hospital, in a conference room separated from the clinic. The interviews were not associated with any clinical context or medical appointment where the participant would be considered as a patient. AS had treated 2/30 of the participants one year prior to the interviews. Each interview was summarized and concluded at the end by the interviewer, and in case of any ambiguities during the interview regarding specific topics, participants could further elaborate their experiences to obtain clarification. The interviews were transcribed verbatim by medical secretaries at the Department of Dermatology and Venereology, Umeå, Sweden.

### Analysis

Qualitative content analysis with an inductive approach towards the data was used as described by Graneheim and Lundman (2004), aiming to find patterns in data through various steps of abstraction leading to a more general and theoretical level of understanding (Graneheim et al., 2017).

The procedure of content analysis as in this study started by reading the text from the interviews several times and identifying the relevant content areas according to our aim. In the next step, units of analysis were condensed, without losing their core meaning. Meaning units (words, phrases, or sentences) corresponding to the aim of this study were further condensed and labelled with codes (Krippendorff, 1980). Codes, sharing similarities, were put together into subcategories defining a specific topic. Subcategories were then abstracted into categories in a higher logical order. A higher order of abstraction is reached by pairing similar categories, and thus one or several themes can be formed (Graneheim et al., 2017). In this study, we identified and presented one theme.

### Rigour

Trustworthiness is an important, and a complex, aspect when using qualitative methods which we addressed through continuous discussions and debriefing between the authors until consensus was achieved (Graneheim & Lundman, 2004). Purposive sampling was used to obtain appropriate and useful information from a selected group of persons affected by PH, thereby increasing the rigour of the study (Campbell et al., 2020). Credibility was ensured by providing a thick description of data and phenomenon. Further, the interviews provided rich data, as a total of 30 men and women were interviewed, which gives large variations in experiences. However, no new major experiences regarding PH were expressed in the final interviews. In addition, suitable quotes illustrating the experiences were used in presentation of the results to strengthen credibility of the analysis. To increase dependability, there was an ongoing reflection and discussion within the research group throughout all steps of the analysis until consensus was achieved. This was conducted because higher levels of abstractions increase one’s influence on data and could present a challenge to the findings (Graneheim et al., 2017).

### Ethical considerations

The study was approved by the Regional Ethical Review Board, Northern Sweden, in May 2016, Decision No. 2016-242-32 M. Participants’ rights to autonomy, informed consent, and confidentiality were respected. All participants were given both written and oral information about the study and written consent was obtained at the time of the interview.

## Results

The emerging theme in this study, *Navigating in the fog*, highlights the experiences of men and women living with PH in their search for help because of excessive sweating. This metaphor not only emphasizes the uncertainty and concern of the participants suffering from PH, but also the uncertainty shown by some health care professionals (HCPs) regarding excessive sweating. While the participants were often uninformed about their medical condition and struggled to find more knowledge about the disease, HCPs could sometimes hinder the participants from getting treatment rather than providing evidence-based care. Related categories and corresponding subcategories revealed differing aspects of how encounters with HCPs, poor knowledge, and support could act as barriers or facilitators in the search for help and treatment. While the path to obtaining an effective treatment was easier for some, others were delayed in their efforts and had suffered from PH for many years. Despite the delay in receiving appropriate help, support of others or changes in life circumstances made it possible to navigate the hurdles described by the participants in finding the information and treatment needed. A presentation of categories and related subcategories with illustrative quotes from interviews follows below.

### Doubtful encounters with health care professionals

This category describes various aspects of uncertainty and dissatisfaction experienced when seeking contact with health care services. The category embraces the following subcategories, which each describe negative aspects of the consultations: *Encounter ignorant health care professionals* and *Worry about being denied available treatment*.

#### Encounter ignorant health care professionals

Health care professionals’ acknowledging and understanding problems associated with excessive sweating was important in the general view of the participants. It was reported that HCPs did not acknowledge or understand the presented symptoms and related problems, when participants summoned the courage to seek help. Not being listened to or being dismissed was described as discouraging and deprecatory. The participants were often provided with explanations regarding their sweating as temporary or caused by normal hormonal changes during puberty. Practical advice from HCPs on how to address their excessive sweating was often based on general beliefs, rather than on scientific evidence, which were perceived as unhelpful.

*The physician was shocked about my problems, as I was from the encounter. I was given advice that anyone could have given, for example, use a tissue and dry yourself*. (Male participant #5)

#### Worry about being denied effective treatment

After getting confirmation from health care services that the excessive sweating was abnormal and should be assessed for treatment, a new concern arose for some participants. Now, the problem was acknowledged, but were the symptoms profound enough to justify coverage of treatment costs? Not qualifying for treatment—excessive sweating could be associated with cosmetic problems—or not having severe enough sweat production were described as deterring the participants from further help-seeking, as it was indicated that nothing could be done regarding their problems. Other sources causing anxiety and concerns were relapses after a period of treatment as the effect diminished, having a preferred timing for treatment, and wishing that treatment sessions were given according to the needs of patients.

*If I could wish for something, it would be that the treatment would be scheduled longer ahead at the end of spring, because it is always worrisome not knowing*. (Male participant #1)

### Helpful encounters with health care professionals

This category describes different aspects of interactions between HCPs and the participants, which were found helpful and contributed to life changing events. To be met with understanding, empathy and an open-minded and listening attitude was relieving for the participants. Further, referral to specialist care and treatment with botulinum toxin was liberating and enabled new possibilities in life. The following subcategories describe the positive aspects of the consultations: *Meet knowledgeable health care professionals* and *Receive successful treatment*.

#### Meet knowledgeable health care professionals

Participants who achieved confirmation of their symptoms and were referred to a specialist described satisfaction that they had been listened to and that their excessive sweating was confirmed as more than normal. Some HCPs were perceived as unaware of what the participants were suffering from, but as curious about the problems excessive sweating created. This initiated the process of obtaining treatment, as the HCP referred the participant for specialist assessment. Having sweat marks on clothes or sweating during the consultation also visualized and strengthened the participant’s case. The systematic approach by some HCPs towards the medical history of the participants regarding heredity, earlier treatment failures, and how the disease affected their daily lives was highly appreciated; being referred to specialist care for further treatments was also described as satisfying.

*I spoke to my doctor and felt that she really listened. She had heard about the condition, but was not sure. She did some research and referred me to another specialist. I was very satisfied when I left her office*. (Female participant #4)

#### Receive successful treatment

Receiving treatment with botulinum toxin removed sweat production, but most importantly, it removed the shame of visible sweat during social interactions. Not having to think about the social consequences of sweating was described as having a profound effect on the well-being and health of the participants. Being able to adopt a more relaxed posture by not hiding body parts such as hands or armpits and choosing attire without thinking about its camouflage qualities were positive changes in life. In some, the treatment was described as liberating, and it was expressed that the state of the participants’ mental health could have been better if they had received treatment years ago. One participant described how the reduced sweating after treatment with botulinum toxin had relieved the pain from arthritis in her hands, and it was difficult to recall the hardships she had to endure prior to the treatment.

*After the first treatment when I sat on the bus home, my hands were all warm, and I cried. I cried of gratitude and happiness that, finally, this was the first time my hands felt really warm*. (Female participant #8)

### Delays due to stigma and inadequate knowledge

This category describes aspects such as the sweating causing shame and stigma preventing the participants to reveal their problems to others and difficulties in finding information not only about excessive sweating in general but also among HCPs. The following subcategories describe the participants’ struggle with feeling shame and not finding the needed information they sought about PH as reasons delaying them seeking help from health care services: *Feel embarrassed to reveal problems caused by sweating, Resign in help seeking due to personal lack of information* and *Demand knowledge about the condition in society as well as in health care*.

#### Feel embarrassed to reveal problems caused by sweating

The excessive sweating caused embarrassment and shame. Hence, participants revealed that they hid their sweating and were uncomfortable revealing their condition to anyone, even to their closest family members. Discussing the issue of excessive sweating and related problems with medical professionals or the closest family was difficult and anguished for some participants, which ultimately contributed to delays in care seeking. Some participants also remarked on the perceived risk of being exposed if they were to have made an appointment for a consultation with an HCP. It would have raised questions among their family or friends, which was not a risk worth taking.

*It feels embarrassing talking to the school nurse when you are in a sensitive and young age. If they had had a brochure about the disease, I would have just looked at that instead. (*Female participant #14)

#### Resign in help seeking due to personal lack of information

Not being able to understand what the excessive sweating represented, combined with unavailable information about the condition, created and cemented a sense of resignation that persisted for years. Participants had been given explanations such as personality traits or physiological changes during puberty that could have caused a temporary increase in sweating. These repeated explanation models from parents or HCPs served as a foundation for the belief that the symptoms would eventually reside one day. Even though some participants were aware that they were sweating notably more than peers, they still tried to normalize their condition and did not believe that their symptoms represented a pathological condition.

*If it is something I do not know anything about, then why should I go to the doctor? What can they do regarding an unknown condition if not even I, being affected, know anything more about sweating*? (Male participant #14)

#### Demand knowledge about the condition in society as well as in health care

Participants described inadequate knowledge about PH within health care and also in the general community, specifically in forums such as schools and pharmacies and among family members. Relatives, friends, and school staff were often surprised or indifferent when participants informed them about their excessive sweating. They wished for openness within society regarding PH and its treatment to diminish the shame and ease the burdens of excessive sweating. Suggestions were given on how to improve awareness about PH by educating physicians and nurses in primary health care centres, providing pamphlets with information at health care establishments or pharmacies, and sharing information about hyperhidrosis by school nurses and school welfare officers.

*One way to remove the mark of shame from the disease is to talk about it. I would like it if physicians, nurses, and others could speak about the disease when encountering young individuals with symptoms*. (Female participant #8)

### Supported urge for help

This category describes how the participants’ help seeking was facilitated by the support of those in their proximity and the role that different media platforms provided by sharing information regarding excessive sweating. These different aspects are described in the following subcategories: *Supported by others to seek medical help, Knowledge about options through media*, and *Need change due to personal life circumstances*.

#### Supported by others to seek medical help

Friends, colleagues, or relatives who acknowledged the problems were able to play an important role by giving insights and support on how to proceed when seeking help. While some relatives and friends shared their own experiences about treatment, others offered practical help by further investigating what could be done within their social network. These efforts encouraged the participants to be more active in seeking alleviation from health care services. Participants who had children showing similar symptoms of PH as themselves became more aware regarding the suffering of their offspring. By seeking help on their behalf, those participants were able to explore what could be done and were also able to take part in the care provided for their children.

*My daughter, who is 12 years old, has the same problems as I have. I thought there must be a connection here. So, I started asking questions. What can we do about it? That is the thing—it is your daughter and you want to help her*. (Female participant #6)

#### Knowledge about options through media

Getting information regarding excessive sweating by searching Internet forums, blogs, websites of private clinics, and articles in magazines was a way for participants to seek and increase their knowledge about PH. However, in trying to navigate online for information, the participants were often unfamiliar with medical terminology, and reliable sources of information about the disease. This research was described as a difficult task, and they often by chance succeeded in finding what they were looking for. Finding information through these sources also indicated that there were others with the same problems. After finding the initial information, participants would seek complementary sources to increase their knowledge about PH or contact health care services to discuss the possibility of receiving available treatments.

*I first checked Google for information, then looked at YouTube about how the treatment was done. (Female participant #3)*

#### Need change due to personal life circumstances

Changes in life, such as moving out from the childhood home or performing frequent social activities such as speaking in front of others, for example, at university, were described as a turning point. Specific events such as getting married or dating new partners were other reasons for seeking help. Additionally, having to wear work uniforms or representing an organization dealing with clients exacerbated the sweat problems and something had to be done about it. Disease burdens piling up over time or a severe sweat attack with great impact on work or private life were also mentioned as reasons to contact health care and seek help.

*In the end, my cup was full. I had thought about it (treatment) so many times. That there must be something that can be done for my sweat problems*. (Male participant #7)

## Discussion

To our knowledge this is the largest qualitative study of health care seeking among 30 adult men and women diagnosed with PH. The aim of this study was to describe the experiences of men and women with PH when seeking help within health care services for their condition. Our findings highlighted the blurred maze of barriers and facilitators to be navigated while dealing with the shame of PH in order to receive needed care and treatment. The findings further emphasized how ignorance and neglect from HCPs downgraded participants and withheld diagnosis and treatment. Furthermore, HCPs who listened, confirmed the condition, and provided treatment contributed to a positive change in the daily life of their patients. Thus, the metaphoric theme summarizing the experiences of men and women with PH in this study was *Navigating in the fog*.

Two of our categories (*Helpful encounters with health care professionals* and *Doubtful encounters with health care professionals*) described the experiences of the participants in their encounters with HCPs. The nature of this encounter was decisive, as HCPs who listened to and had a systematic approach of investigating the symptoms were described as successful in providing help. In today’s health care, the mental, physical, and social health of an individual is a complex matter. Reducing care to a single symptom could create inequality and selection bias for those seeking help. In PH, HCPs with a holistic view regarding the consequences of excessive sweating rather than an emphasis on the production of sweat, will be able to better understand and acknowledge the shame, psychological stress, and social restrictions experienced by the patients.

The delay in receiving help due to PH was described in one of our categories, relating to difficulties in finding information in public forums and from health care services, also reported by Kamudoni et al. (2017). Pamphlets about PH in schools or other forums, as suggested by the participants, could increase awareness regarding hyperhidrosis and facilitate help-seeking. Providing access to information about PH is important since it has been described that uncertainties and deficient knowledge in adolescents add to the stigma associated with PH (Trettin et al., 2020). Considering adults with PH, an assumption could be made that the neglect from HCPs could further stigmatize and delay their process in obtaining help. Comparing diseases is a difficult task, and a remark in delay while seeking help for visible and stigmatizing conditions often recurs. For example, in obesity, health care providers with a negative attitude have been reported by patients causing hinderance in further help-seeking (Fruh et al., 2016). While women suffering from hirsutism, rejected or humiliated by their physician, are described as more vulnerable and they abstain from seeking help as their condition deteriorates (Ekbäck et al., 2011). Individuals with PH need and expect HCPs to listen to them so that they can make their situation understood. Thus, being affirmed regarding difficulties experienced from PH, could lead to a positive spiral of reducing delay in obtaining help, reducing stigma from hyperhidrosis and positively influence patients in coping with a chronic disease as PH is.

The World Health Organization (WHO) has declared that health services have to be available, accessible, acceptable, and of adequate quality (AAAQ, 2016). Undiagnosed and untreated PH with hidden psychological traumas and stigma could have negative financial and mental impact on those affected. Data regarding socioeconomic impacts in individuals with PH does not exist. It could be argued that sweating could interfere with occupation, leading to sick leave or less effectiveness at work, increasing cost for public health through consultations, expenses for unhelpful remedies, and a reduced well-being affecting persons in their family. In PH, the largest gain for the patients, according to AAAQ, would be to raise the quality of healthcare. This can be done by educating HCPs in identifying individuals with PH and providing effective treatments. Educational efforts such as incorporating PH into lectures and seminars for general practitioners, residents in family medicine, and medical and nursing students, would spread more knowledge about hyperhidrosis through the medical professions. Further studies are needed in the future to investigate the effect of such measures.

In summary, PH is a condition that has received little attention in clinical practice as well as in research. The experiences of affected persons are briefly mentioned as sub-findings in existing studies, and qualitative studies are lacking. This study describes the experiences of individuals diagnosed with PH when seeking healthcare and suggests improvements that could benefit patients with hyperhidrosis.

### Strengths and weaknesses

A major strength of this study was the vivid and expressive interviews of both men and women, where participants generously shared their variety of positive and negative experiences, despite being aware that the researchers also work in health care. Another strength was examination of the results, which were analysed and reviewed by individuals with different perspectives and relations to PH: a medical student (FG), a clinician working with PH patients (AS), and researchers with various medical backgrounds at the Department of Nursery, Umeå University (MP, CB) and Department of Dermatology and Venereology, Umeå University (EN). This enabled us to mitigate the risk of perception bias, since in qualitative content analysis the same material can yield different results for different persons if awareness and precautions are not constantly checked.

There may be an ethical dilemma as the interviewer (AS) had treated two of the participants for PH one year prior to the interviews. As PH is a small research field within dermatology, preconceived knowledge of the subject may be an advantage as long as it does not affect the informants or the interpretation of the results. Having met AS beforehand may have contributed to an openness in the interviews but also created an obligation to partake. To minimize any potential bias in this situation, we emphasized that participation was voluntary, and questions which participants felt uncomfortable to answer could be left without comments. In addition, to ensure trustworthiness, co-workers came from various disciplines and were all involved in the whole research process where potential biases and findings were discussed to the point of consensus.

## Conclusions and implications

A holistic approach towards patients with PH is important to reduce stigma and acknowledge the problems that are encountered in their daily lives. By emphasizing how the disease affects the quality of life rather than investigating symptom severity, medical consultations could produce higher patient satisfaction and the possibility of patients being offered treatment options. Educating HCPs during their basic training and making information available about PH in schools and pharmacies could improve the general knowledge of PH and facilitate the path to receiving help for excessive sweating. Primary care providers could also focus on increasing literacy regarding PH among their staff, so that this patient category can be identified and assessed without delay.
